# Familial Hyperparathyroidism

**DOI:** 10.3389/fendo.2021.623667

**Published:** 2021-02-25

**Authors:** Jenny E. Blau, William F. Simonds

**Affiliations:** ^1^ Early Clinical Development, Cardiovascular, Renal and Metabolism, BioPharmaceuticals R&D, AstraZeneca, Gaithersburg, MD, United States; ^2^ Metabolic Diseases Branch, National Institute of Diabetes and Digestive and Kidney Diseases, National Institutes of Health, Bethesda, MD, United States

**Keywords:** tumor suppressor, oncogene, multiple endocrine neoplasia, MEN1, jaw tumor syndrome, CASR, CDC73, GCM2

## Abstract

Regulation of the serum calcium level in humans is achieved by the endocrine action of parathyroid glands working in concert with vitamin D and a set of critical target cells and tissues including osteoblasts, osteoclasts, the renal tubules, and the small intestine. The parathyroid glands, small highly vascularized endocrine organs located behind the thyroid gland, secrete parathyroid hormone (PTH) into the systemic circulation as is needed to keep the serum free calcium concentration within a tight physiologic range. Primary hyperparathyroidism (HPT), a disorder of mineral metabolism usually associated with abnormally elevated serum calcium, results from the uncontrolled release of PTH from one or several abnormal parathyroid glands. Although in the vast majority of cases HPT is a sporadic disease, it can also present as a manifestation of a familial syndrome. Many benign and malignant sporadic parathyroid neoplasms are caused by loss-of-function mutations in tumor suppressor genes that were initially identified by the study of genomic DNA from patients who developed HPT as a manifestation of an inherited syndrome. Somatic and inherited mutations in certain proto-oncogenes can also result in the development of parathyroid tumors. The clinical and genetic investigation of familial HPT in kindreds found to lack germline variants in the already known HPT-predisposition genes represents a promising future direction for the discovery of novel genes relevant to parathyroid tumor development.

## Introduction

Typically evidenced by elevated serum calcium, primary hyperparathyroidism (HPT) is a disorder of mineral metabolism caused by the inappropriate or excessive secretion of parathyroid hormone (PTH) from one or several abnormal parathyroid glands (1). The majority of cases of HPT are sporadic and arise or occur randomly with no apparent predisposition (~95%). With respect to the remaining < 5% of patients with a familial predisposition to develop HPT, most carry germline mutation of a gene known to confer susceptibility to parathyroid tumor development (listed in **Table 1**). Even though these disorders are rare, study of the molecular genetics of these uncommon familial HPT syndromes has provided considerable insight into the molecular pathophysiology of both sporadic and familial parathyroid neoplasia. Since the release of parathyroid hormone (PTH) from parathyroid chief cells is tightly regulated by the calcium-sensing receptor (CASR), a cell surface-expressed G protein-coupled receptor (GPCR) belonging to GPCR family C (2), mutation in the germline of *CASR* or other genes transducing and propagating the CASR signal can also result in heritable syndromes associated with hypercalcemia and PTH levels that are high or inappropriately normal. This chapter will review and summarize current knowledge of the molecular pathophysiology and clinical genetics of familial syndromes that predispose to parathyroid gland neoplasia and HPT.

**Table 1:** Hereditary Hyperparathyroidism.

Disorder	Gene	Gene location	Mechanism	Clinical Features
**Syndromic HPT**				
**Multiple Endocrine Neoplasia (MEN)**				** **
**MEN Type 1**	*MEN1*	11q13	TS; LoF	Multigland hyperparathyroidism, functional and/or non-functional pancreatic NET and anterior pituitary adenomas. Associated features include foregut NET, cutaneous lipomas, collagenomas, facial angiofibromas; esophageal, uterine leiomyoma
**MEN Type 2A**	*RET*	10q11.21	PO; GoF	Medullary thyroid carcinoma, bilateral pheochromocytoma; hyperparathyroidism due to parathyroid adenoma
**MEN Type 4**	*CDKN1B*	12p13.1	TS; LoF	Multigland hyperparathyroidism; pituitary adenoma; reproductive organ tumors (e.g., testicular cancer, neuroendocrine cervical carcinoma); adrenal & renal tumors
**Hyperparathyroidism-Jaw Tumor Syndrome (HPT-JT)**	*CDC73 (formerly HRPT2)*	1q31.2	TS; LoF	Single- or multigland hyperparathyroidism with an increased predisposition to parathyroid carcinoma; cemento-ossifying fibromas of the maxilla or mandible (“jaw tumors”); Wilms tumor, renal MEST; uterine pathology including adenomyosis and leiomyoma
**Isolated HPT**				
**Familial Hypocalciuric hypercalcemia (FHH)**				
**FHH type 1**	*CASR*	3q13.3 - q21.1	ICSST; LoF	Lifelong elevation of serum calcium with inappropriately low urinary calcium excretion (FeCa < 1%) and normal or mildly elevated PTH
**FHH type 2**	*GNA11*	19p13.3	ICSST; LoF	
**FHH type 3**	*AP2S1*	19q13.32	ICSST; LoF	
**Neonatal Severe Primary Hyperparathyroidism (NSHPT)**	*CASR*	3q13.3-q21.1	ICSST; LoF	Severe hypercalcemia at birth with associated bone demineralization and failure to thrive (associated with bi-allelic LoF of *CASR*)
**Familial isolated hyperparathyroidism (FIHP)**				Multigland hyperparathyroidism
** GCM2**	*GCM2*	6p24.2	PO; GoF	Multigland hyperparathyroidism (enriched in Ashkanazi Jewish populations)

HPT, primary hyperparathyroidism; TS, tumor supressor gene, LoF, loss of function; NET, neuroendocrine tumor; PO, proto-oncogene; GoF, gain of function; MEST, mixed epithelial and stromal tumor [of the kidney]; ICSST, impaired calcium sensing or signal transduction.

## The Pathophysiology and Clinical Presentations of Primary Hyperparathyroidism

Mammals, like all vertebrates, depend on calcium for diverse cellular processes such as the actions of critical calcium-dependent enzymes, neuromuscular excitability, muscular contraction, intracellular second messenger signaling, neurotransmitter release, and membrane permeability. The ability of many proteins to reversibly bind calcium ions, which enables signaling events to be registered through the presence or absence of such binding (and associated protein conformational changes), is facilitated by calcium’s particular coordination chemistry (3). A system of physiologic calcium homeostasis, that depends on calcium reserves present in bone, ensures the continued normal functioning of the myriad intracellular processes that vitally depend on calcium.

In order to maintain the ambient calcium concentration within a defined physiologic range, parathyroid hormone (PTH) secretion from the parathyroid glands is tightly regulated in response to changes in the circulating ionized calcium level. Calcium sensing is mediated by the CASR, a GPCR situated on the plasma membrane of chief cells in the parathyroid glands and a critical regulator of PTH secretion (4, 5). In the bone, PTH binds to specific PTH1R receptors present on the surface of osteoblasts, stimulating the expression of receptor activator of nuclear factor kappa-B ligand (RANKL), which in turn recruits and activates osteoclasts whose bone resorptive activity mobilizes calcium from bone thus raising the ambient ionized calcium concentration (6). In another classic endocrine negative feedback loop, 1,25-dihydroxyvitamin D, the active form of cholecalciferol whose biosynthesis in proximal renal tubular cells is stimulated by PTH, inhibits the biosynthesis and release of PTH from parathyroid cells (7–10). A widely accepted clinical definition of HPT is the concurrent demonstration of hypercalcemia and an elevated or inappropriately normal PTH (1). The great majority of parathyroid tumors are adenomas (i.e. benign neoplasms), with parathyroid carcinoma making up less than 1% of cases of HPT in most clinical series.

Hypophosphatemia is a frequent concomitant of HPT. HPT figures prominently in the differential diagnosis of hypophosphatemia and the coincidence of hypercalcemia and hypophosphatemia is highly suggestive of HPT (11). The mechanism of relative or absolute hypophosphatemia in HPT involves the actions of PTH on PTH1R receptors present in the proximal renal tubule that promote the urinary excretion of phosphate. Activation of PTH1R by PTH downregulates the surface expression of type II sodium phosphate cotransporters (NPTIIa, SLC34A1; NPTIIc, SLC34A3) present on the apical membranes of proximal renal tubule cells resulting in PTH-stimulated phosphaturia and renal phosphate wasting (12, 13).

There is a wide spectrum of clinical presentations of HPT (1). This reflects the broad range of actions of PTH in target tissues as well as the myriad potential signs and symptoms of hypercalcemia *per se*. The metabolic abnormality is asymptomatic in as many as 70–80% of patients, often discovered incidentally on routine blood chemistry panels. Sporadic primary HPT occurs at all ages, but peaks in the sixth decade of life and is more common in women, with a female-to-male ratio between 2:1 and 3:1. Common symptomatic manifestations include fatigue, weakness, cognitive changes such as difficulty concentrating and impaired memory, gastrointestinal changes such as constipation and bloating, polyuria, nephrolithiasis, and osteoporosis. While the severity of symptoms may be proportional to the degree of hypercalcemia, for any degree of hypercalcemia, older patients tend to be more sensitive to the cognitive and neuromuscular manifestations. When severe, the renal phosphate wasting and subsequent hypophosphatemia associated with HPT may contribute to patients’ fatigue and weakness. Advanced HPT is classically characterized by osteitis fibrosa cystica, a syndrome of severe skeletal demineralization associated with cyst-like “brown tumors”, bone pain or tenderness, bone fractures, and skeletal deformities such as bowing of weight-bearing bones (14).

Parathyroid carcinoma is a rare cause of HPT, seen in fewer than 1% of sporadic cases (15, 16). Parathyroid carcinoma can be challenging to diagnose, as many of the histopathologic features are neither specific nor sensitive. Histopathologic findings include fibrous bands, elevated mitotic index, nuclear atypia, invasion of neighboring tissues, and perineural or angio-lymphatic invasion. Not infrequently the diagnosis is made only retrospectively, years after the initial parathyroid surgery, subsequent to the clinical manifestation of local or distant metastases. Clinical findings suggestive of carcinoma include a palpable neck mass, hoarseness (which may be indicative of vocal cord paresis and laryngeal nerve damage), serum calcium greater than 14 mg/dL, and overt bone and/or kidney disease at the time of presentation.

While most cases of HPT are sporadic, inherited forms of HPT represent approximately 2 to 5% of cases. As exemplified in **Table 1**, research into the genetics and molecular pathophysiology of the smaller subcategory of familial cases has nonetheless provided important insights regarding the genes and pathways that contribute to parathyroid tumor development. Multiple endocrine neoplasia type 1 (MEN1), the hyperparathyroidism-jaw tumor syndrome (HPT-JT), familial isolated hyperparathyroidism (FIHP), and multiple endocrine neoplasia type 2A (MEN2A), are the most frequently encountered inherited disorders that predispose to HPT (17–21). Familial hypocalciuric hypercalcemia (FHH), also known as familial benign hypercalcemia, is a related autosomal dominant condition often mis-diagnosed as HPT that is characterized by lifelong asymptomatic hypercalcemia that results from impaired calcium sensing or downstream signal transduction. In FHH, partial or even subtotal parathyroidectomy does not correct the hypercalcemia (22). The relevance of these rare familial disorders to the basic molecular pathogenesis of parathyroid neoplasia will be discussed in more detail in the sections to follow.

## Tumor Suppressor Genes and the “Two-Hit” Hypothesis of Tumor Development

Cellular activation of tumorigenesis requires transformation of normal cell into a neoplastic derivative. This process is typically regulated by genes that encode proteins that help control cell growth and proliferation. Tumorigenesis initiates when the balance between cell growth and inhibition is lost, and genes that positively regulate growth (proto-oncogenes) or inhibit growth (tumor suppressor genes) are either constitutively activated or inactivated, respectively.

The most common mechanism for tumor growth in hereditary tumor syndromes, which account for ~5–10% of all cancer, is inactivation of tumor suppressor genes. Knudson’s “two‐hit hypothesis” has been fundamental for understanding tumor suppressor genes and familial tumor-predisposing syndromes. Before the advancement of molecular genetics, Knudson used mathematical modelling to compare the clinical presentation of sporadic and inherited cases of retinoblastoma (RB) in children to better understand the latency of the disease (23). By evaluating the time at which both eyes would be affected by retinoblastoma if one or two hits to DNA were required, Knudson accurately predicted the relative chance of this occurring. The conclusion that retinoblastoma required two-hits, one from the germline (inherited) and a subsequent second hit to inactivate the gene (termed “loss of heterozygosity” or LOH), was confirmed with the discovery of the *RB* gene in 1986 and identification of LOH in the tumor tissue (24). The most common tumor suppressor genes such as *p53, RB*, and *PTEN* are frequently identified in various tumor syndromes (25). A similar observation of the two-hit hypothesis holds true for inherited causes of hyperparathyroidism due to tumor suppressor genes such as *MEN1* and *CDKN1B* (**Table 1**).

## Proto-Oncogenes and Heritable Oncogenes

Another potential molecular mechanism for tumor development involves mutant genes called oncogenes that drive cell growth. Oncogenes derive from naturally occurring genes, called proto-oncogenes. Typically proto-oncogenes are genes which positively regulate cell division and/or cell growth under normal conditions (26). Examples of proto-oncogenes include *SRC*, *HRAS*, *KRAS*, *NRAS*, *WNT1*, and *MYC*. Oncogenes result from the mutational activation (e.g. *via* the acquisition of mutations that result in constitutive signaling activity) or overexpression of proto-oncogenes that can induce cell division and cell growth, causing tumor formation often in a tissue-specific fashion. The gene products encoded by proto-oncogenes often belong to mitogenic signaling pathways. With respect to the etiologies of currently understood inherited cancer syndromes, germline mutational gain-of-function of proto-oncogenes is rare compared to loss-of-function mutations in tumor suppressor genes (see below). Constitutive mitogenic signaling resulting from the germline gain-of-function of most proto-oncogenes is likely to be incompatible with normal embryonic and fetal development which requires extremely precise regulation of intercellular communication.

## Multiple Endocrine Neoplasia Type 1 Syndrome (MEN1)

The initial identification of the co-occurrence of parathyroid, pancreas, and [anterior] pituitary tumors was published by Harvey Cushing in 1927 in a patient with acromegaly, hyperparathyroidism, and an islet cell tumor (27). Further case series emerged (28, 29) and recognition of the autosomal dominant inheritance of parathyroid hyperplasia, pancreatic islet cell tumors and anterior pituitary tumors was reported in 1954 by Paul Wermer (previously referred by the eponym Wermer’s syndrome) (30). Further understanding of the syndrome led to a hypothesis by Zollinger and Ellison who suspected an ulcerogenic hormone arising from a pancreatic islet cell tumor (31). Additional manifestations of the disease have emerged over time to create a complex picture of neoplasms affecting multiple organs (**Table 1**).

Linking the MEN1 trait to an unknown causative gene on chromosome 11q13 (32) and identification of loss of heterozygosity in the tumor tissue (33) ultimately paved the way for identification of the *MEN1* gene in 1997 by positional cloning (34). Subsequent understanding of the protein product menin has evolved over the past 20 years since the discovery of the gene. However, many of its functions remain elusive. While menin is highly conserved in animal species, the amino acid sequence does not show homology with any known proteins. Menin is a nuclear protein with multiple functions, particularly transcription regulation and chromatin modification (35). In addition, menin functions as a DNA-repair protein in response to DNA damage, cell signaling, cytoskeletal structure, cell division, cell adhesion or cell motility (36).

*MEN1* gene mutations have evolved from inherited tumor syndromes to recognizing the role of somatic *MEN1* mutations in sporadic neuroendocrine tumors (37–39). Advances in tumor genetic profiling of pNETs have identified somatic *MEN1* mutations in 30–44% of sporadic islet cell tumors (38, 40, 41). Key genetic regulators of the mTOR signaling pathway, histone modification, altered telomere length (ALT) and DNA damage repair pathways have also emerged as important pathogenic contributors to somatic pancreatic neuroendocrine tumor (pNET) development. In particular, up to 40% of somatic pNETs have mutations in either the apoptotic regulator *DAXX* (death-domain–associated protein) or the chromatin modifier *ATRX* (α thalassemia/intellectual disability syndrome X-linked) where they promote ALT and chromosomal instability (40, 42, 43). These alternations in *MEN1*, *DAXX* and *ATRX* have led to the hypothesis of molecular subtypes, which may confer both therapeutic interventions and possible survival benefit (40, 44).

MEN1 syndrome typically manifests with early onset of HPT. In known MEN1 kindreds, children and adolescents are recognized to have mild biochemical evidence of hyperparathyroidism with hypercalcemia prior to the onset of symptoms (45, 46). Penetrance of HPT is almost complete by age 50 (47). Nevertheless, some MEN1 patients may become symptomatic by the second decade with non-specific complaints (constipation, fatigue, abdominal pain, etc.) or present with end-organ effects such as nephrolithiasis or fracture (48). Similar to other familial HPT syndromes, parathyroid hyperplasia occurs in all four glands over time with asynchronous and often asymmetric growth (49). In contrast to patients with *CDC73* mutations (as described below), parathyroid carcinoma is exceedingly rare in MEN1.

Epigenetic modulators also have been evaluated and linked to pathogenesis in HPT, although the data remains the strongest with links to parathyroid carcinoma. DNA methylation and chromatin modifications, for example, may contribute to the pathogenesis of HPT, although the MEN1 gene itself does not appear to be subject to methylation changes (50) and parathyroid adenomas do not appear to be affected by global methylation patterns. Rather, parathyroid tumors mainly demonstrate hypermethylation involving the CpG islands of the promoter region of specific genes in processes involved in cell cycle and transcription (e.g. CDKN2A/B, RB, WT1, etc.), Wnt/B-catenin (e.g. APC, SFRP1/2/4) and membrane transporters (e.g. MDR1). It may be that hypermethylation can link the frequency and prognosis (e.g. aggressive and/or malignant) of parathyroid tumors in the future. For a fuller review of this topic, please see the recent study by Corbetta and co-workers (51).

Other epigenetic regulators, including small non-coding microRNA, have also been postulated to contribute to the pathogenesis of tumor initiation among patients with MEN1. MicroRNA is known to contribute to multiple cellular processes, including cell proliferation, adhesion, cell death and differentiation (52) and negatively regulate post-transcriptional gene expression (53). One study evaluated parathyroid adenomas in MEN1 and suggests three main dysregulated microRNA: miR-1301, miR-4258 and miR-664. The latter two microRNAs were down-regulated in parathyroid cells that have lost both the wild type MEN1 alleles while miR-1301 was over-expressed after biallelic inactivation of wild type MEN1 (54). While further work needs to be done to explore the contribution of microRNA and other epigenetic regulators in tumorigenesis of MEN1, epigenetics may play an important role in the differentiation of phenotypic disease burden and may also help further inform diagnosis and prognosis in the future.

**Figure 1:**
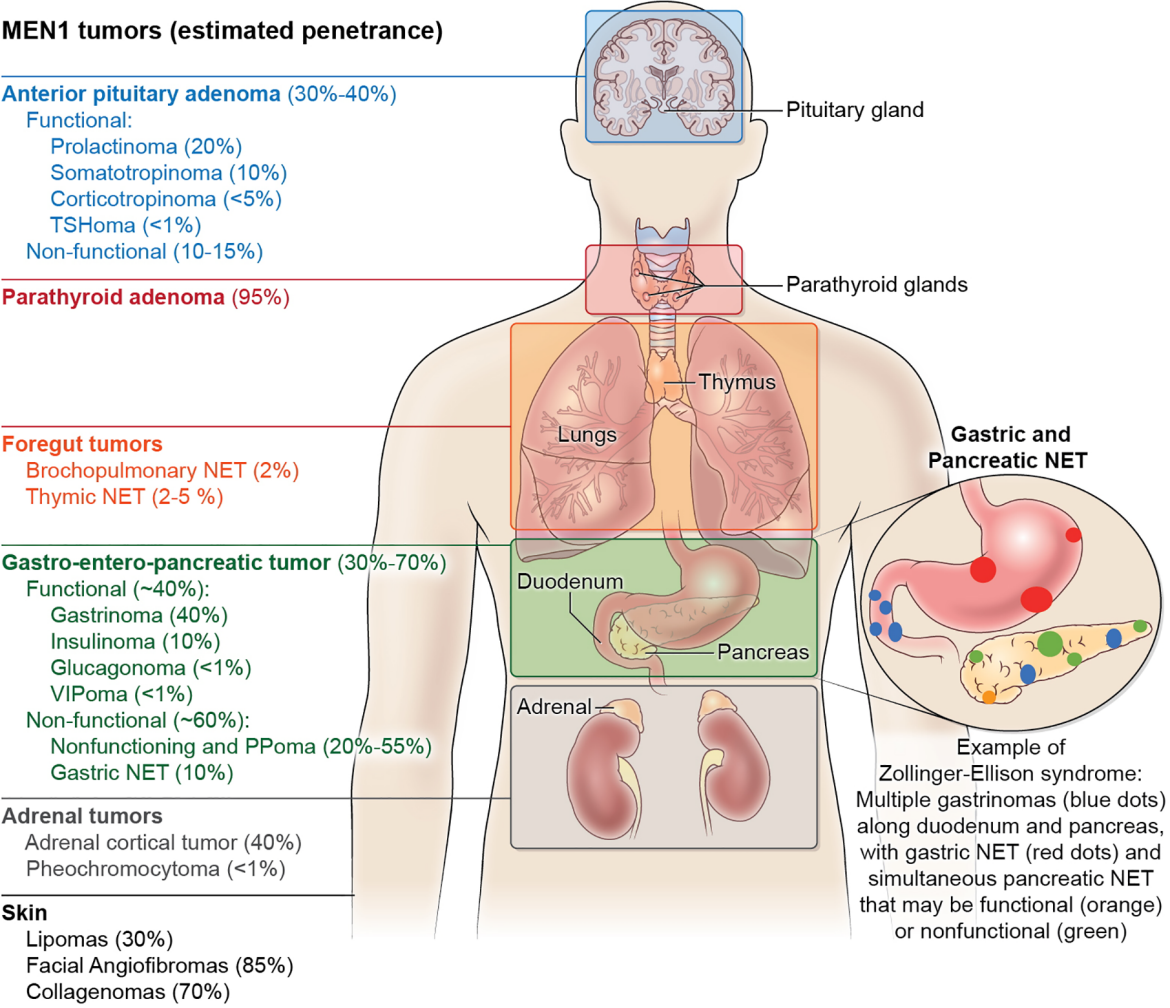
The clinical manifestations and estimated tumor penetrance of multiple endocrine neoplasia type 1 (MEN1) syndrome. The main manifestations of MEN1 include anterior pituitary adenomas, pancreatic neuroendocrine tumors, and primary hyperparathyroidism. Except for multi-gland hyperparathyroidism which approximates 95% penetrance by age 50, the manifestations of other tumors in MEN1 have no genotype-phenotype correlation and cannot be predicted, even within kindreds. Neuroendocrine tumor (NET) hormone secretion in the anterior pituitary and pancreas can be considered functional or non-functional, while duodenal NET and parathyroid adenomas are always functional with the secretion of gastrin and parathyroid hormone, respectively. As pictured, Zollinger-Ellison Syndrome can present with multiple submucosal duodenal gastrinomas with simultaneous pancreatic gastrinomas, non-functional pancreatic tumors, as well as the development of gastric NET typically within thickened gastric folds (not pictured). Less frequent manifestations of MEN1 include foregut bronchopulmonary and thymic neuroendocrine tumors, as well as adrenal cortical adenomas and pheochromocytomas. Skin manifestations of MEN1 include lipomas that can occur anywhere on the body, collagenomas mostly on the trunk, and facial angiofibromas on the nasal bridge and cheeks. Manifestations that have been described but are not pictured include meningiomas, leiomyoma (uterus, esophagus, ureter, bladder), café-au-lait spots, and malignant breast tumors.

Anterior pituitary adenomas in MEN1 may be functional or non-functional. The genetic landscape of pituitary tumors has advanced over the last decade, with the identification of somatic (*GNAS*, *USP8*, *GPR101*) and germline (*MEN1*, cyclin dependent kinase inhibitor genes, *AIP*, *DICER1*, *PRKAR1A*, *PRKACA*, *SDHx*, and *GPR101*) drivers of pituitary tumorigenesis (55). Familial causes of pituitary adenomas account for ~5% of all cases, but should be considered especially in young patients, those with syndromic manifestations or a family history suggesting a germline mutation. Typically, MEN1 associated pituitary adenomas are benign, but may cause significant morbidity in the setting of hormonal hypersecretion, enlargement that impinges on the optic chiasm, pituitary apoplexy, or post-surgical hypopituitarism (56, 57). Prolactinomas represent the most common clinically presenting pituitary tumors, followed by non-functional, somatotroph pituitary tumors, and, rarely, corticotroph or thyrotroph adenomas (47) (**Figure 1**). Interestingly, while no reproducible variant has been identified in MEN1, two large kindreds followed over 30 years demonstrated a frequent prolactinoma and less frequent gastrinoma than typical MEN1 that was reproducible among kindreds and could not be explained by genetic mutation (58). Early and frequent (1–3 years) magnetic resonance imaging (MRI) screening identifies non-functional adenomas that may remain indolent (59).

The third hallmark feature of MEN1 includes gastroenteropancreatic neuroendocrine tumors (GEP-NET). Up to 80% of MEN1 patients have radiological evidence of GEP-NETs. Other complex hereditary syndromes also manifest particularly with pNETs, including >15% of patients von Hippel–Lindau disease (VHL), <10% of patients with neurofibromatosis 1 (NF1) and <1% of patients with tuberous sclerosis (60). The most common functional pancreatic NET in MEN1 is a gastrinoma, which can also occur in the duodenal mucosa as small multifocal tumors and leads to the Zollinger-Ellison Syndrome (ZES) (61) (**Figure 1**). About 40–60% of pNETs will secrete polypeptide hormones such as gastrin, insulin, glucagon or vasoactive intestinal peptide (VIP), while the others are non-functional. Hormonal excess induces clinical symptomatology that may occur simultaneously. The spectrum of symptoms includes multiple severe ulcers of the stomach and duodenum (ZES), hypoglycemia with seizures (insulinoma), glucose intolerance and watery diarrhea with hypokalemic alkalosis (WDHA) syndrome (VIPoma), or mass effect symptoms (62). The introduction of proton pump inhibitors in the late 1980s significantly shifted the mortality curve from gastrinoma-induced gastric ulcer perforation to non-functional pancreatic neuroendocrine tumors with unpredictable malignant potential. It is now recognized that thymic NETs, a rare but aggressive tumor, carry the highest odds ratio of death among MEN1 patients (63). Nevertheless, given the rarity of these thymic tumors, pancreatic NET are the main focus of therapeutic interventions to improve MEN1 mortality (64–66).

Several guidelines to assist the clinical practitioner in the management of patients with MEN1 have been published (47, 67). These aim to establish guiding principles for the evaluation, treatment, and genetic testing of patients with MEN1 and their family members.

## Multiple Endocrine Neoplasia Type 2A (MEN2A)

MEN2A is an inherited endocrine cancer syndrome that includes HPT as one of its less penetrant and clinically less consequential features. Besides primary HPT, MEN2A is characterized by the predisposition to develop medullary thyroid cancer (MTC), a neoplasm of thyroid parafollicular C cells, and pheochromocytoma which, in the context of MEN2A, is typically benign and often bilateral (68). Occasionally patient with MEN2A develop cutaneous lichen amyloidosis. In the clinical setting of MEN2A, HPT resembles sporadic HPT and is usually mild and due to benign parathyroid disease. MEN2A is inherited in an autosomal dominant fashion and results from germline activating missense mutation in the *RET* proto-oncogene on the long arm of chromosome 10. The *RET* proto-oncogene encodes a receptor tyrosine kinase that binds, together with co-receptor glycosylphosphatidylinositol-anchored protein Gfra1 (69), members of the glial cell line-derived neurotrophic factor (GDNF) family of extracellular signaling molecules.

Germline gain-of-function mutations of the *RET* proto-oncogene are associated with three separate heritable endocrine neoplasia syndromes, all of which are associated with MTC: MEN2A also known as Sipple syndrome, multiple endocrine neoplasia type 2B (MEN2B) also known as the mucosal neuroma syndrome, and familial medullary thyroid cancer (FMTC). Usually parathyroid tumors and HPT are not part of the disease spectrum of MEN2B or FMTC. Distinct patterns of disease result from particular RET mutations giving rise to apparent genotype-phenotype correlations. Approximately 95% of MEN2A cases result from the presence in the germline of nonsynonymous codon substitutions affecting the cysteine-rich region within the RET receptor’s large extracellular domain, namely missense mutations of *RET* codons 609, 611, 618, 620, or 634 (70). Indeed, approximately 85% of cases of MEN2A are caused by germline missense substitution of RET residue cysteine-634 (68, 71). Somatic mutations in the *RET* proto-oncogene associated with MEN2 rarely cause sporadic parathyroid tumors (72, 73).

Based on the consensus view among an international group of endocrinologists and other expert practitioners, guidelines for the clinical management of patients with MEN2 have been published (67). The purpose of these guidelines is to establish principles for the evaluation, indications and timing of surgical intervention, and interpretation of the genotype-phenotype correlates of patients with MEN2 and others in their kindreds.

## Multiple Endocrine Neoplasia Type 4 (MEN4)

Pellegata and coworkers first described MEN4 in a multi-generational kindred with manifestations overlapping those of MEN1 but whose affected members lacked germline *MEN1* mutation (74, 75). A germline heterozygous stop-gain mutation in the cyclin dependent-kinase inhibitor p27(Kip1), encoded by *CDKN1B*, was identified in the proband with acromegaly and HPT and in several other members of this kindred (74). Genetic analysis of rats with a multi-tumor syndrome phenotype called multiple endocrine neoplasia X (MENX) had previously drawn attention to the *Cdkn1b* locus (74, 76). The rat MENX phenotype was discovered accidently when some rats in a Sprague–Dawley colony were observed to spontaneously develop multiple endocrine tumors (77). The MENX phenotype in rats was manifested by parathyroid hyperplasia, multifocal anterior pituitary adenoma, adrenal and extra-adrenal pheochromocytoma, thyroid C-cell hyperplasia, and pancreatic islet cells hyperplasia. MENX in rats was recessively inherited and caused by a frameshift mutation in *Cdkn1b* (74, 76). In the study by Pellegata et al., only the proband among members of the MEN4/MENX kindred described was reported to have HPT (74).

Prior to 2019, reports of kindreds demonstrating segregation of a mutation in *CDKN1B* with an MEN4 phenotype across multiple generations were quite limited. Several groups investigated for a possible role for *CDKN1B* mutation in parathyroid neoplasia following the original report by Pellegata et al. (74). Several studies examined *MEN1* mutation-negative patients and kindreds expressing MEN1-like tumors and harboring germline mutation in *CDKN1B* and which thus could be considered as MEN4 (74, 78–85). Apart from the verification of HPT in association with *CDKN1B* mutation in a pair of monozygotic twins (79), none of these reports prior to 2019 had described kindreds with more than one individual with HPT proven to segregate with the *CDKN1B* mutation. In 2019 however, a report by Frederiksen et al. described a large multi-generational Danish MEN4 family in which HPT occurred in 13 members and segregated with a germline frameshift *CDKN1B* mutation (86). Molecular genetic analysis of sporadic parathyroid adenomas demonstrating that *CDKN1B* mutation can be both somatic and clonal further supports the characterization of *CDKN1B* as a gene predisposing to the development of primary parathyroid tumors (87, 88). A recent study suggests that germline *CDKN1B* mutation can also present as apparently sporadic, isolated pediatric Cushing’s disease (89).

## The Hyperparathyroidism-Jaw Tumor Syndrome (HPT-JT)

HPT-JT was first recognized as a form of familial HPT that was genetically distinct from MEN1 and MEN2A in 1990 by Gene Jackson and co-workers (90). Central to this recognition was re-evaluation of a family, first described three decades earlier (91), in which occurrence of multiple ossifying fibromas of the maxilla and mandible in two affected members of the third generation was seen to be similar to the jaw tumors of four of five affected members of the first generation. As noted originally by Jackson and co-workers (90), the maxillary and mandibular tumors seen in HPT-JT are distinct from the “brown tumors” of HPT (often seen in the context of *osteitis fibrosa cystica* and metabolically severe HPT), because the former can appear and/or enlarge in affected members in the absence, or following surgical correction of, HPT. Furthermore, whereas the “brown tumors” of HPT can occur anywhere in the axial or appendicular skeleton, the jaw tumors found in HPT-JT kindreds are restricted to the maxilla and mandible. The jaw tumors in HPT-JT are histologically distinct fibro-osseous lesions without the abundant multinucleated giant cells seen in “brown tumors,” and have been formally classified as cemento-ossifying fibromas (92). The exact cell of origin from which cemento-ossifying fibromas derive in HPT-JT is not precisely known, but they are generally believed to be mesodermal odontogenic tumors derived from the mesenchymal blast cells of the periodontal ligament, with the potential to form fibrous tissue, cementum, and bone or a combination thereof (93, 94).

HPT-JT is an autosomal dominant inherited syndrome with variable penetrance and expressivity. HPT is the most penetrant feature of HPT-JT and is the manifestation that most often brings carriers to medical attention. Besides HPT, the key clinical features of HPT-JT include cemento-ossifying fibromas restricted to the maxilla and mandible (as described above), renal lesions, and uterine tumors in women (90, 95–97). In contrast to sporadic HPT and MEN1, parathyroid cancer is relatively frequent in the context of HPT-JT, and affects ~20% of those with HPT (90, 95, 96, 98, 99).

In the preponderance of HPT-JT kindreds, a germline inactivating mutation of the *CDC73* gene (formerly called *HRPT2*) on the long arm of chromosome 1 can be identified (19, 100) The *CDC73* gene encodes parafibromin, a protein of 531 residues, that is considered to be a tumor suppressor protein because germline mutation predicted to cause loss-of-function predisposes to the neoplastic expressions of HPT-JT (100). The majority of germline *CDC73* mutations in kindreds with HPT-JT are predicted to inactivate gene function *via* frameshift or nonsense mutation, with only a minority of the variants encoding missense mutations (99, 101). Partial or complete deletion of the *CDC73* gene has also been described in patients and kindreds with HPT-JT (102–105).

The high frequency of parathyroid cancer is a hallmark of HPT-JT. A genotype-phenotype correlation has been observed among *CDC73*-mutation carriers such that those with frameshift, nonsense or deletion mutations are nearly 7-fold more likely to develop parathyroid cancer than patients harboring missense *CDC73* mutations (99). In apparently sporadic cases of parathyroid cancer, mutations of *CDC73* are frequently identified (106–110). Interestingly, germline loss-of-function mutation in *CDC73* may be found in some 25% of patients with seemingly sporadic parathyroid carcinoma, suggesting that such patients may have either *de novo* germline mutation in *CDC73* or else a *forme fruste* of HPT-JT (19, 107, 108).

A potential genotype-phenotype correlation has also been observed with respect to certain renal manifestations of HPT-JT. Mixed epithelial and stromal tumor of the kidney (MEST), a rare type of renal tumor, has been associated with HPT-JT and/or *CDC73* germline mutation in at least two kindreds (111, 112). MEST is a renal neoplasm, characterized by cystic structures lined by epithelium and admixed with ovarian-type stroma, that must be diagnosed differentially from cystic hamartoma of the renal pelvis, adult type mesoblastic nephroma, and leiomyomatous renal hamartoma (113, 114). MEST in the context of HPT-JT and/or *CDC73* germline mutation appears to correlate with a specific *CDC73* missense mutation in which the initiator methionine of parafibromin is replaced with isoleucine, i.e. the Met1Ile genotype (100, 111, 112). Wilms tumor has been identified in members of several families with HPT-JT (115, 116). Renal cysts are also reported to be part of the clinical spectrum of HPT-JT and *CDC73* germline mutation (19). No genotype-phenotype correlation has been reported for Wilms tumor and renal cysts in the context of *CDC73* germline mutation.

The presence of uterine manifestations of HPT-JT was first elucidated by Bradley et al. who recognized a high frequency of menorrhagia often leading to early hysterectomy among affected women and adult female carriers (95). There was a range of uterine pathology among women with HPT-JT who underwent hysterectomy that included adenosarcomas, adenofibromas, leiomyomas, adenomyosis, and endometrial hyperplasia (95). The uterine manifestations of HPT-JT significantly reduced the reproductive fitness of affected women. Lifelong monitoring for uterine tumors with routine gynecologic care and pelvic ultrasound examination as clinically indicated has been recommended for women with HPT-JT, starting at reproductive age (19).

**Figure 2:**
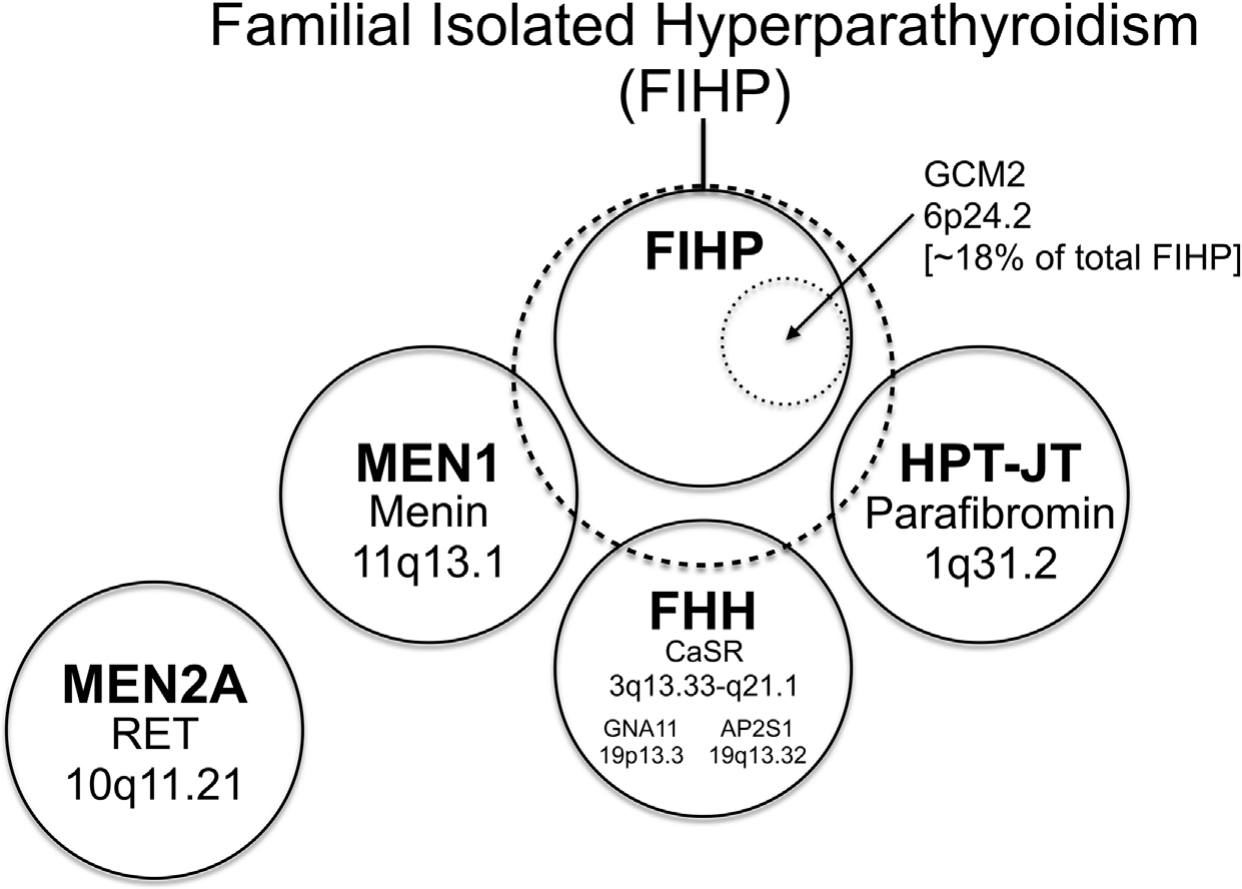
The relationship among familial forms of hyperparathyroidism that may present as familial isolated hyperparathyroidism (FIHP) as a Venn diagram. The large dashed circle represents the set of patients that can present with a provisional diagnosis of FIHP at the time of initial ascertainment. This includes patients with FIHP who have been evaluated for, but lack findings diagnostic of, MEN1, FHH, and HPT-JT (nonsyndromic FIHP; in a solid circle). Approximately 18% of nonsyndromic FIHP kindreds harbor germline gain-of-function mutations in *GCM2* (inner dotted circle) (see text), whereas the remainder have currently unknown genetic etiologies. Subsets of patients with incomplete expression of MEN1, FHH and HPT-JT (the total set of patients in each syndrome represented by a solid circle) can also present with the FIHP phenotype (and thus overlap with the large dashed circle). The distinction between the nonsyndromic FIHP category and the syndromic categories arbitrarily depends on the depth and rigor of evaluation and the sensitivity of diagnostic tests used to detect the syndrome, testing that can include germline gene mutational analysis. MEN2A is a familial form of hyperparathyroidism that seldom if ever presents as FIHP, with patients usually coming to medical attention for signs and symptoms of medullary thyroid cancer and/or pheochromocytoma. Within each circle representing a defined syndrome are included the genetic locus (or loci in the case of FHH; see text) of the syndromic trait and the associated gene product. The causative gene for HPT-JT that encodes parafibromin is *CDC73*, formerly called *HRPT2*. The relationship among the patient sets illustrated as circles in this diagram is intended to be qualitative and neither the area of each circle nor the area of overlap between circles has any quantitative significance.

A subset of kindreds classified as FIHP have been shown to harbor germline *CDC73* mutation, suggesting that incompletely penetrant HPT-JT can phenocopy FIHP (see below and **Figure 2**). Approximately 20% of obligate or genetically confirmed *CDC73* mutation-positive subjects lack any clinical manifestations of HPT-JT at the time of kindred ascertainment (99), in line with the variable penetrance and expressivity of *CDC73* mutation. Lifelong surveillance of initially asymptomatic *CDC73* mutation carriers is recommended since the penetrance of the manifestations of HPT-JT increases with age (117).

## Familial Isolated Hyperparathyroidism (FIHP)

As is evident from its appellation, FIHP defines a category of familial HPT that encompasses kindreds containing two or more members with HPT but that lack other, specific features of a familial HPT syndrome: MEN1, MEN2A, HPT-JT or FHH (**Figure 2**) (118). FIHP is a diagnosis of exclusion with the strength of the diagnosis proportional to the depth and rigor of clinical and genetic evaluation. Following the initial ascertainment and clinical evaluation of possible FIHP kindreds, genetic testing may reveal that germline mutation of *MEN1*, *CDC73*, or *CASR* accounts for a fraction of the FIHP kindreds under investigation (20, 119–122). Nevertheless, following careful clinical and genetic analysis, the majority of FIHP kindreds have been shown to lack germline mutation in the established HPT-susceptibility genes (**Figure 2**) (20, 119, 123). Because sporadic HPT is not uncommon (1), some small kindreds with a tentative diagnosis of FIHP may in fact reflect the coincidental clustering of sporadic cases among close relatives.

Genetic variants that result in missense mutations in GCM2, which is a nuclear transcription factor required for parathyroid gland organogenesis named for its homology to the “glial cells missing” (gcm) gene in *Drosophila*, were recently discovered in the germline DNA of eight different families with FIHP (21). This gene had already been implicated in parathyroid gland dysfunction, since it had been previously shown that germline loss-of-function and dominant-negative mutations of GCM2 were associated, respectively, with autosomal recessive and autosomal dominant familial isolated hypoparathyroidism (124, 125). Testing *in vitro* has shown that the two germline variants in *GCM2* associated with FIHP act as gain-of-function mutations (21). The FIHP-associated missense mutations in GCM2 map to its C-terminal conserved inhibitory domain (CCID) and increase its transcriptional activity, presumably through a mechanism of dis-inhibition, suggesting that in the context of FIHP *GCM2* is a parathyroid proto-oncogene. Specific variants affecting the CCID of *GCM2* are enriched among FIHP kindreds and sporadic HPT patients with an Ashkenazi Jewish background (126). Genetic screening of multiple FIHP kindreds suggests that approximately 18% harbor germline activating GCM2 mutations (21), leaving ~80% of carefully curated FIHP families without a currently-defined genetic basis (118). Clinical investigators in other centers have also documented rare germline *GCM2* variants that map to the CCID domain and segregate with the disease in a subcategory of FIHP kindreds (127). Activating germline variants in *GCM2* that map to the CCID domain have also been found in low frequency among patients with typically-presenting sporadic HPT who underwent parathyroid tumor excision and appear to be of low penetrance (128).

## Familial Hypocalciuric Hypercalcemia (FHH)

FHH describes a condition of PTH-dependent hypercalcemia, resembling and in the differential diagnosis of HPT, that is typically benign (**Table 1**) (22, 129). The condition, also known as “familial benign hypercalcemia”, is genetically heterogeneous and results from mutations that cause parathyroid gland insensitivity to extracellular calcium with a resulting rightward shift of the set point for suppression by calcium of PTH secretion. As a result of this intrinsic insensitivity of the parathyroid cells to suppression by circulating calcium, affected patients from FHH kindreds almost always remain hypercalcemic following partial or subtotal parathyroidectomy. FHH is inherited in an autosomal dominant manner and usually results in mild hypercalcemia, non-suppressed or mildly elevated PTH, and relative hypocalciuria. The hypercalcemia observed in FHH is highly penetrant across the age spectrum, including in neonates and infants (22, 130). The majority of cases of FHH results from heterozygous germline loss-of-function mutation of the calcium-sensing receptor, encoded by the *CASR* gene on the long arm of chromosome 3 (4, 131). FHH due to inactivating mutation in the *CASR* gene is classified as type 1 FHH (FHH1) and is the most common type (132). Neonatal severe primary hyperparathyroidism (NSHPT) that presents as severe hypercalcemia, typically occurring in the first 6 months of life, is a rare autosomal recessive disorder that most often is a consequence of the compound heterozygous or homozygous inheritance of two mutationally-inactivated *CASR* alleles (133). While true parathyroid tumors demonstrate cellular monoclonality, the hyperfunctioning parathyroid tissue removed from a patient with NSHPT demonstrated generalized polyclonal hyperplasia by molecular genetic analysis, illustrating the non-neoplastic quality of the abnormal parathyroids that result from *CASR* loss-of-function mutation (134).

Reduced cell surface expression of the CASR protein has been demonstrated in parathyroid adenomas and may explain the rightward shift of the calcium set point and the impaired calcium-mediated suppression of parathyroid hormone release typical of such adenomas. Decreased *CASR* transcript expression, but not loss-of-heterozygosity at the *CASR* chromosomal locus, has been demonstrated in benign parathyroid tumors (135). In molecular genetic studies of sporadic parathyroid tumors reported to date, somatic inactivation of the *CASR* gene has not been described (136, 137).

A minority of families with FHH result from germline mutations in genes other than CASR on chromosome 3q. Type 2 FHH (FHH2) results from inactivating mutation in the germline of the G protein α11 subunit encoded by *GNA11* (138, 139). *GNA11* encodes the α-subunit of the signal-transducing G protein downstream of the CASR (140). The CASR allosteric activator cinacalcet has been reported to correct the hypercalcemia of patients with FHH2 due to *GNA11* mutation (141). Type 3 FHH (FHH3) results from germline loss-of-function mutation in adaptor related protein complex 2 subunit sigma 1 (AP2S1), an adaptor protein involved in clathrin-mediated endocytosis and encoded by *AP2S1* on chromosome 19q (142–145). In FHH3 the vast majority of inactivating mutations in AP2S1 involve missense substitutions of codon Arg-15, but analysis of large scale exome datasets suggests that other residues may be more rarely involved (146). Mutant forms of AP2S1 involving codon Arg-15 that result in FHH3 may exert dominant-negative effects on CASR signaling (144). In molecular genetic analyses of sporadic parathyroid tumors, somatic loss-of-function mutation in the genes encoding GNA11 and AP2S1 have thus far not been described.

## Differential Diagnosis of Familial Forms of Hyperparathyroidism

When a familial form HPT is suspected based on patient or family medical history, and/or one or more suspicious non-parathyroid features, certain key clinical or laboratory findings can help in the differential diagnosis. A history of pituitary adenomas, pNETs, bronchial carcinoid, or duodenal endocrine tumors in the patient or first-degree relatives favor MEN1. Suspicion of MEN2A is heightened if a history of MTC or pheochromocytoma is documented in the patient or a family member. History of parathyroid cancer, maxillary or mandibular tumors, Wilms tumor, or uterine abnormalities requiring early hysterectomy in the patient or another member of the kindred, should elevate the possibility of HPT-JT. Persistent hypercalcemia following parathyroidectomy, hypocalciuria, hypermagnesemia, and hypercalcemia in family members younger than 10 years of age could be clues to the diagnosis of FHH. Genetic testing, conveniently performed with a gene panel that includes *MEN1, CDC73, CASR, GNA11, AP2S1, CDKN1B*, and *GCM2*, can help clarify the diagnosis and establish the genetic etiology. Recommendations for the testing of younger patients with seemingly sporadic HPT are described below.

## Discussion

Even though inherited forms of HPT represent only a small proportion of total cases (< 5%), investigation into the molecular basis of these rare familial syndromes has resulted in considerable insight into the genetics and pathophysiology of both sporadic and familial HPT and spotlighted the importance of genes such as *MEN1, CDC73, CASR, GNA11, AP2S1, CDKN1B*, and *GCM2*. It seems very likely that gain- or loss-of-function mutation of other genes, currently unrecognized, can also promote parathyroid tumor formation. As an illustration of this, the risk predisposing to the development of parathyroid tumors in most FIHP kindreds appears to result from the germline mutation of genes currently not recognized to play a role in parathyroid neoplasia. This inference follows from the fact that nearly 70% of families initially characterized with an FIHP phenotype in several clinical studies that screened for germline *MEN1*, *CASR*, and *CDC73/HRPT2* gene mutation, were found to have no currently recognized syndromic or genetic basis (**Figure 2**) (20, 119–121). From the FIHP families which are *MEN1*, *CASR*, and *CDC73/HRPT2* mutation-negative, estimates are that approximately 20% carry germline gain-of-function mutations in the *GCM2* proto-oncogene (21). The clear implication is that some 80% of FIHP families have no currently defined genetic etiology for their predisposition to HPT.

The analysis of DNA extracted from parathyroid tumors using methods such as comparative genomic hybridization (CGH) to highlight particular chromosomal regions that have lost or gained segments of DNA also implies the presence of currently unidentified parathyroid tumor suppressors and oncogenes. Several research groups have demonstrated recurrent DNA loss at chromosomal locations 1p, 6q, 9p, and 13q in parathyroid tumors, indicating the possible presence there of currently unrecognized parathyroid tumor suppressor genes (147–150). On the other hand, the demonstration of specific chromosomal gain at chromosomal loci 9q, 16p, 19p, and Xq in benign or malignant parathyroid tumors suggests the possible presence at these loci of novel parathyroid oncogenes (147, 149–151).

What clinical, demographic, or historical considerations should persuade the practitioner to screen individual patients for a genetic basis for their HPT, even if they lack obvious features of a syndrome? As was discussed above in the case of tumor suppressor genes, germline mutation predisposes to earlier onset of disease so very often it is the younger cohort of seemingly sporadic patients with HPT who are considered for such genetic screening. Skandarajah and co-workers performed a retrospective genetic analysis of 21 patients who had undergone surgery for HPT before the age of 40. Although none had suspicious personal or family histories suggestive of MEN1, one patient, who had a double parathyroid adenoma at surgery, was found to have a germline *MEN1* frameshift mutation (152). In another study from Carling and colleagues, 86 patients ≤ 45 years of age with seemingly sporadic HPT underwent genetic analysis for genes predisposing to HPT. Eight of the 86 patients (~ 9%) were found to harbor germline loss-of-function mutations in known HPT susceptibility genes: four *MEN1*, three *CASR*, and one *CDC73* (153). An expert panel of physicians, surgeons, and geneticists assembled to provide clinical practice guidelines for MEN1 suggested that gene mutational testing be performed on patients presenting with seemingly sporadic HPT younger than 40 years of age due to multi-gland parathyroid disease (47). A recent retrospective study of 121 patients screened for familial forms of HPT found that with respect to sole risk factors, a positive family history, but not age at diagnosis or the finding of multiglandular parathyroid disease, was strongly predictive of a pathogenic germline variant in a HPT susceptibility gene (132).

## Author Contributions

JB and WS conceived, planned, and executed the writing of this chapter. All authors contributed to the article and approved the submitted version.

## Funding

The Intramural Research Program of the National Institute of Diabetes and Digestive and Kidney Diseases (ZIA DK043012-18) supported this research.

## Conflict of Interest

JB is employed by the company AstraZeneca.

The remaining author declares that the research was conducted in the absence of any commercial or financial relationships that could be construed as a potential conflict of interest.

The handling editor declared a past co-authorship with the authors JB and WS.
